# Quantitative and Confirmatory Analysis of Pesticide Residues in Cereal Grains and Legumes by Liquid Chromatography–Quadrupole-Time-of-Flight Mass Spectrometry

**DOI:** 10.3390/foods10010078

**Published:** 2021-01-03

**Authors:** Shizuka Saito-Shida, Satoru Nemoto, Hiroshi Akiyama

**Affiliations:** Division of Foods, National Institute of Health Sciences, Tonomachi 3-25-26, Kawasaki-ku, Kawasaki, Kanagawa 210-9501, Japan; nemoto@nihs.go.jp (S.N.); akiyama@nihs.go.jp (H.A.)

**Keywords:** pesticides, liquid chromatography–quadrupole-time-of-flight mass spectrometry, multiresidue method, cereal grains, legumes

## Abstract

For controlling pesticide residues in food and ensuring food safety, multiresidue methods that can monitor a wide range of pesticides in various types of foods are required for regulatory monitoring. In this study, to demonstrate the applicability of liquid chromatography–quadrupole time-of-flight mass spectrometry (LC–QTOF-MS) for quantitative and confirmatory analysis of pesticide residues in cereal grains and legumes, the LC–QTOF-MS method using full-scan acquisition was validated for 151 pesticides in brown rice, soybeans, and peanuts at a spiked level of 0.01 mg/kg. With the exception of 5 out of 151 target pesticides, sufficiently high signal intensities were obtained at 0.005 μg/mL (corresponding to 0.01 mg/kg). Trueness was in the range 70–95%, with intra- and inter-day precisions below 16% and 24%, respectively, with the exception of 7 pesticides in brown rice, 10 pesticides in soybeans, and 9 pesticides in peanuts. No interfering peaks were observed near the retention times of the target pesticides. Furthermore, information on accurate fragment-ion masses obtained by a data-independent acquisition enabled unambiguous confirmation. The results suggest that the LC-QTOF-MS method is suitable for pesticide residues’ analysis of cereal grains and legumes, and can be utilized for regulatory routine analysis.

## 1. Introduction

Pesticides are used worldwide to increase crop yields by protecting crops from pests, including insects, rodents, fungi, and weeds; however, the intake of pesticide residues contained in foods may adversely affect human health [1]. To ensure food safety and protect consumer health, international organizations such as the Codex Alimentarius Commission, established by the Food and Agriculture Organization of the United Nations (FAO) and the World Health Organization (WHO), and the European Union (EU), as well as many individual countries, including Japan, have established maximum residue limits (MRLs) to regulate pesticide residue levels in foods. In Japan, MRLs are currently established for various foods with respect to more than 750 agricultural compounds, i.e., pesticides, veterinary drugs, and feed additives. Therefore, the need for multiresidue methods, which detect a wide range of pesticides in various types of foods, is increasing in laboratories concerned with the regulatory monitoring of pesticide residues.

Nowadays, liquid chromatography (LC) and gas chromatography (GC) coupled with triple quadrupole mass spectrometry (MS/MS) operated in selected reaction monitoring (SRM) mode are the most widely used techniques for analyzing pesticide residues in foods. They are highly sensitive and selective, which enables the robust quantification of trace amounts of pesticide residues in complex matrices. In recent years, LC and GC coupled with high-resolution mass spectrometry (HR-MS) methods, such as time-of-flight (TOF)-MS, quadrupole-TOF-MS (QTOF-MS), Orbitrap-MS, and quadrupole-Orbitrap-MS (QOrbitrap-MS) have also been employed for the screening and quantification of pesticide residues [2,3,4,5,6,7,8,9,10,11,12,13,14]. LC and GC coupled with HR-MS operating in full-scan mode with high mass accuracy have several advantages over LC–MS/MS and GC–MS/MS operating in SRM mode: (1) There are no limits on the number of target compounds that can be analyzed simultaneously [7,8]. (2) The optimization of MS parameters, for example, SRM transitions, cone voltage, or collision energy, for individual analytes is not needed [8,14]. (3) The adjustment of retention time windows for the target analytes is not required even if the mobile phase or analytical column is changed. (4) The methods allow retrospective analysis for nontarget or unknown compounds by reprocessing previously acquired data without re-injection of the samples [15,16,17]. Furthermore, hybrid HR-MS, such as QTOF-MS and QOrbitrap-MS, offer fragment-ion information, which could be used for confirmation purposes [2,11,18]. Accordingly, numerous methods based on LC or GC coupled with HR-MS have been published recently for analyzing pesticide residues in vegetables and fruits [3,4,5,8,9,10,11,13,14]. In our previous work, we reported the quantitative analyses of pesticide residues in tea [19] using LC–QTOF-MS and LC–Orbitrap-MS. However, to the best of our knowledge, few papers have reported the application and validation of LC coupled with HR-MS for the quantitative analysis of pesticide residues in cereal grains and legumes, such as rice, soybeans, and peanuts. Cereal grains and legumes comprise complex matrices, containing high amounts of lipids and/or starch, which can potentially interfere with the analyses and cause matrix effects. Therefore, they are considered to be difficult matrices for the analysis of trace amounts of pesticide residues [20].

The aim of the current study is to evaluate the applicability of LC–QTOF-MS for the quantitative analyses of pesticide residues in cereal grains and legumes containing high amounts of lipids and/or starch. Brown rice, soybeans, and peanuts are selected as representative foods, and the LC–QTOF-MS method is validated for 151 pesticides at a concentration of 0.01 mg/kg. In addition, data-independent acquisition (DIA) is carried out to obtain information regarding the fragment ions, and to demonstrate the capability of LC–QTOF-MS for confirmative analyses.

## 2. Materials and Methods 

### 2.1. Reagents and Chemicals

Pesticide analytical grade toluene and acetonitrile, LC-MS grade water, and methanol were obtained from Kanto Chemical (Tokyo, Japan). Diatomaceous earth (Celite^®^ 545), analytical grade ammonium acetate, dipotassium hydrogen phosphate, potassium dihydrogen phosphate, and pesticide analytical grade sodium chloride were purchased from FUJIFILM Wako Pure Chemical (Osaka, Japan). 

Pesticide standards, except for aramite and etrimfos, were procured from Hayashi Pure Chemical (Osaka, Japan), Kanto Chemical, FUJIFILM Wako Pure Chemical, Dr. Ehrenstorfer (Augsburg, Germany), Riedel-de Haën (Seelze, Germany), and Sigma-Aldrich (St. Louis, MO, USA). Stock standard solutions of each pesticide were prepared in acetonitrile or methanol, depending on their solubility, at a concentration of 1 mg/mL. Standard solutions (100 μg/mL in methanol) of aramite and etrimfos were obtained from AccuStandard (New Haven, CT, USA). A mixed standard solution (1 μg/mL) was prepared by mixing the stock standard solutions and diluting with acetonitrile.

Leucine–enkephalin, used as a reference compound in LC–QTOF-MS analyses, was obtained from Waters (Milford, MA, USA). A 1-μg/mL leucine–enkephalin standard solution was prepared in methanol/water (1:1, *v/v*). 

### 2.2. Materials

Brown rice and soybeans were purchased from a local market in Tokyo (Japan), and peanuts cultivated in Chiba (Japan) were obtained via the Internet. Brown rice and soybeans were ground using a centrifugal mill (Ultra Centrifugal Mill ZM 200; Retsch, Haan, Germany). Peanuts were milled using a laboratory mill (SCM-40A, Shibata, Japan).

Tandem graphitized carbon black (GCB)/primary secondary amine (PSA) cartridges (InertSep GC/PSA, 500 mg/500 mg) were bought from GL Sciences (Tokyo, Japan) and octadecylsilyl silica gel (ODS) cartridges (Mega Bond Elut C18, 1000 mg) were purchased from Agilent Technologies (Palo Alto, CA, USA). 

### 2.3. Apparatus

LC–QTOF-MS analyses were performed using an Acquity UPLC I-class system (Waters) coupled to a Xevo G2-S QTOF mass spectrometer (Waters). The chromatographic separation was carried out using an Inertsil ODS-4 column (100 × 2.1 mm, 2 μm; GL Sciences). The mobile phases consisted of 5 mmol/L ammonium acetate in water (A) and 5 mmol/L ammonium acetate in methanol (B). The mobile phase was pumped at a flow rate of 0.3 mL/min with the following gradient profile: 5% B followed by increasing B to 95% at 10 min and holding it at this concentration for 3 min, increasing to 100% at 13.01 min and holding for 5 min, and finally, returning to 5% at 18.01 min. The column temperature was set to 40 °C. The injection volume was 3 μL. The retention times of the target pesticides are presented in Table 1. 

The QTOF mass spectrometer was operated in resolution mode, providing a resolving power of >30,000 at full width at half maximum (FWHM), at *m/z* 556.2766. The following MS conditions were used: ionization mode, electrospray ionization in positive mode (ESI(+)); scan range, *m/z* 50–1000; source temperature, 120 °C; desolvation gas temperature, 450 °C; capillary voltage, 1000 V; cone voltage, 20 V; collision energy, low energy (4 eV) and high energy (ramp from 10 to 40 eV); desolvation gas (nitrogen), 800 L/h; cone gas (nitrogen), 50 L/h; collision gas, argon. Leucine–enkephalin (*m/z* 556.2766) was used as a reference compound, being introduced from a lock spray probe during analyses. The mass window of ±5 mDa was used for the extraction of chromatograms for each target pesticide. The calculated exact mass and retention time for each pesticide are summarized in Table 1.

### 2.4. Sample Preparation

Samples were prepared according to the official Japanese multiresidue method, namely, “Multi-residue Method I for Agricultural Chemicals by LC-MS (Agricultural Products),” except for the use of a tandem GCB/PSA cartridge instead of a GCB/aminopropylsilyl silica gel (NH_2_) cartridge for cleanup.

A 10.0 g sample was weighed in a glass tube and water (20 mL) was added; subsequently, it was left to stand for 30 min. Acetonitrile (50 mL) was added to the mixture; then it was homogenized using a homogenizer (Polytron PT 10–35 GT; Kinematica, Lucerne, Switzerland) for 1 min. The homogenate was filtered with suction, and then the residue was rehomogenized with acetonitrile (20 mL) before being filtered with suction. The filtrates were combined, and the resulting volume was adjusted to 100 mL by the addition of acetonitrile.

A 20 mL aliquot of the extract was added to a 50 mL polypropylene (PP) centrifuge tube containing sodium chloride (10 g) and phosphate buffer (pH 7.0, 0.5 mol/L). The mixture was shaken for 5 min by a shaker (SR-2w; Taitec, Saitama, Japan) and centrifuged for 5 min at 3000 rpm (Centrifuge 8100, Kubota, Japan). The resultant acetonitrile layer was loaded onto an ODS cartridge, which was preconditioned with acetonitrile (10 mL), and then eluted with acetonitrile (5 mL). The resultant eluates were combined and concentrated to approximately 0.5 mL by a rotary evaporator (NVC-2100/N-1000, Eyela, Tokyo, Japan) at <40 °C; it was then dried by evaporation under a nitrogen stream. The residue was redissolved in acetonitrile/toluene (3:1, *v*/*v*, 2 mL) and loaded onto a GCB/PSA cartridge, which was preconditioned with acetonitrile/toluene (3:1, *v*/*v*, 10 mL) and then eluted with acetonitrile/toluene (3:1, *v*/*v*, 20 mL). The eluate was concentrated to approximately 0.5 mL by a rotary evaporator at <40 °C and evaporated to dryness under a nitrogen stream; finally, the resultant residue was redissolved in methanol (4 mL) prior to LC–QTOF-MS analysis.

### 2.5. Method Validation

The LC–QTOF-MS method was validated using a nested experimental design for brown rice, soybeans, and peanuts. Samples were spiked in duplicate at a level of 0.01 mg/kg, and the recovery experiments were repeated on five different days. To prepare the spiked samples, a 1 mL aliquot of the 0.1 μg/mL mixed standard solution was added to 10.0 g of sample, and the mixture was allowed to stand for 30 min before proceeding with the subsequent sample preparation steps. The quantification was carried out using six-point calibration curves with solvent-based standard solutions prepared in methanol. The concentrations of the standard solutions used to construct the calibration curves, to allow quantification, were 0.00125, 0.0025, 0.00375, 0.005, 0.00625, and 0.0075 μg/mL. The linearity of each calibration curve over a wider range was examined in the range of 0.002–0.1 μg/mL.

Matrix-matched standards were prepared by evaporating a 100 μL aliquot of blank solution under a nitrogen stream and then redissolving it in 100 μL of the mixed standard solution in methanol. Matrix effects were evaluated by comparing peak areas of the matrix-matched standards with standards in solvents as follows: average peak area (*n* = 5) of matrix-matched standard/average peak area (*n* = 5) of standard in solvent.

## 3. Results and Discussion

### 3.1. Optimization of LC–QTOF-MS Conditions

A total of 151 LC-amenable pesticides, which had molecular weights from 189 to 746, were selected as target pesticides for this study. Because most of the target pesticides produced high-intensity signals under positive-mode operation of the instrument (c.f., negative-mode operation), and since the instrument used in this study was unable to simultaneously operate in both the positive and negative modes, LC–QTOF-MS analyses were carried out only in the positive mode, using the MS parameters optimized in a previous study [14]. The calculated exact mass of each pesticide is presented in Table 1. Quantification was performed by operating in full-scan acquisition mode using ions with the highest intensity among [M+H]^+^, [M+Na]^+^, and [M+NH_4_]^+^. For most of the target pesticides, the highest intensity was obtained for [M+H]^+^; only 10 compounds were observed to have their highest intensity for [M+NH_4_]^+^ and none of the compounds were seen at their highest intensity for [M+Na]^+^. The mass window for extracting the chromatograms of each pesticide was optimized by comparing the repeatability of the peak areas of the target compounds, obtained by replicate analyses (*n* = 5, 0.01 μg/mL) for the extraction of mass windows of ±2.5, ±5, and ±10 mDa. It should be noted that, in general, a narrow mass window for the extraction of chromatograms will result in low background noise and allow the discrimination of coeluting matrix components. This will increase sensitivity and selectivity; however, the use of a disproportionately narrow window will result in peak shape deterioration and low repeatability. Hence, mass windows of ±5 and ±10 mDa resulted in relative standard deviations (RSDs) of <5% for all the target pesticides; whereas the RSD values for 10 pesticides were >5% with a mass window of ±2.5 mDa. In addition, narrow mass windows produced higher signal-to-noise (S/N) ratios. Therefore, considering these results, the mass window was set to ±5 mDa, as a trade-off between S/N and peak area repeatability.

### 3.2. Method Validation

As mentioned earlier, the samples were prepared according to the official Japanese multiresidue method “Multi-residue Method I for Agricultural Chemicals by LC-MS (Agricultural Products)” prior to analysis by LC–QTOF-MS, except for the modification in the cleanup step. A tandem GCB/PSA cartridge was used instead of a tandem GCB/NH_2_ cartridge for cleanup because the PSA sorbent can more effectively remove acidic matrix components, such as organic acids and fatty acids, compared to a NH_2_ sorbent. The LC–QTOF-MS method was validated in terms of linearity, matrix effect, trueness, intra- and inter-day precisions, and selectivity for detection of the spiking at a concentration level of 0.01 mg/kg with 151 pesticides of brown rice, soybeans, and peanuts. Quantification was carried out using solvent-based calibration curves in this study.

Injecting a standard solution of 0.005 μg/mL, which corresponds to 0.01 mg/kg, five pesticides, i.e., hexaconazole, isoprocarb, methidathion, pentoxazone, and quizalofop ethyl, exhibited insufficient sensitivities, i.e., S/N < 10. Among them, the low sensitivity of quizalofop ethyl could be a consequence of a high background noise level due to polysiloxane contamination, which has a similar calculated exact mass (*m/z* 373.0981, [C_10_H_31_Si_4_^30^SiO_5_]^+^). Therefore, the validation method was continued for 146, of the original 151, pesticides and achieved the required sensitivity of 0.005 μg/mL (corresponding to 0.01 mg/kg). Furthermore, because ferimzone and tricyclazole were detected at concentrations of 0.02 mg/kg and <0.01 mg/kg, respectively, in the brown rice sample used for the method validation in this study, ferimzone and tricyclazole were also excluded from the target compounds for method validation in brown rice. It should be noted that the residue levels of ferimzone and tricyclazole detected in brown rice were below the MRLs (2 ppm and 3 ppm, respectively) established in Japan. 

The results of the recovery experiments are shown in Table 2. The trueness of the target pesticides was in the range of 70 to 120% and within the acceptable range of the criteria required by the Japanese [21] and EU [22] method validation guidelines, except for the cases of 7 pesticides in brown rice, 10 pesticides in soybeans, and 9 pesticides in peanuts. The intra- and inter-day precisions (expressed as RSD) were in most cases <10%. All target pesticides that achieved satisfactory trueness values fulfilled the precision criteria of the Japanese validation guideline, namely <25% for intra-day and <30% for inter-day precisions at 0.01 mg/kg [21]. Calibration curves for the target pesticides in the concentration range 0.00125–0.0075 μg/mL demonstrated sufficient linearity, with coefficients of determination (*r*^2^) of >0.99, with the exception of the five pesticides (hexaconazole, isoprocarb, methidathion, pentoxazone, and quizalofop-ethyl) for which the detection sensitivity was deemed to be insufficiently high. In addition, calibration curves were also linear in the wider range 0.002 to 0.1 μg/mL with *r*^2^ > 0.99, except for the cases of the aforementioned five pesticides. These five pesticides resulted in linear calibration curves in the range 0.01 to 0.1 μg/mL with *r*^2^ > 0.995.

It is well known that LC-MS/MS with ESI is susceptible to ion suppression, especially in complex food matrices, mainly due to the competition between analyte and coeluting matrix components [23]. Because the LC–QTOF-MS analyses were conducted using ESI in this study, ion suppression might also have occurred during these measurements. Thus, matrix effects were evaluated by comparing the peak areas of the matrix-matched standard solution at 0.005 μg/mL (corresponding to 0.01 mg/kg) to those of the standard solution prepared in methanol at the same concentration. The matrix effect values are shown in Table 3 and ranged from 0.8 to 1.1 for 134 (out of 144), 141 (out of 146), and 142 (out of 146) pesticides in brown rice, soybeans, and peanuts, respectively. The results indicate that no significant matrix effect occurred for most of the target pesticides studied, even though the soybean and peanut samples contained high amounts of lipids. Thus, these results suggested that the low trueness values for the acrinathrin (peanuts), cycloprothrin (brown rice), deltamethrin (peanuts), epoxiconazole (brown rice), fenpropathrin (brown rice), fipronil (brown rice), fluvalinate (brown rice, soybeans, and peanuts), hexythiazox (soybeans), imibenconazole (brown rice), and spinosyn A (brown rice and peanuts) samples were mainly caused by ion suppression.

**Figure 1:**
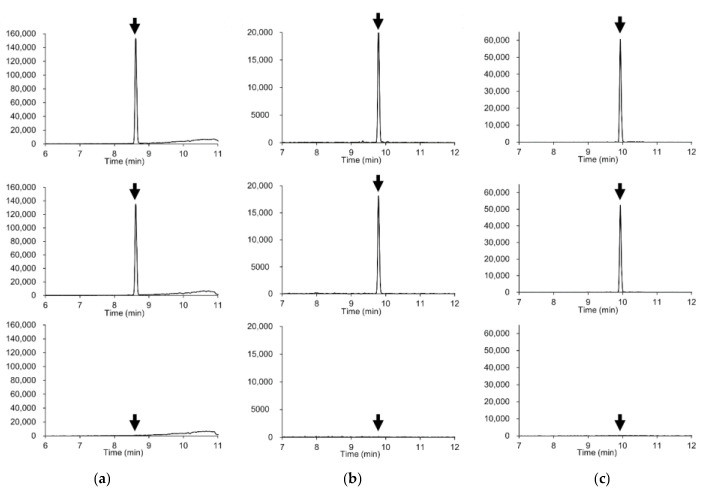
Extracted ion chromatograms (mass window ±5 mDa) of representative compounds. (**a**) Azoxystrobin (*m/z* 404.1241); (**b**) Diazinon (*m/z* 305.1083), (**c**) Indoxacarb (*m/z* 528.0780). Upper plots: standard solution in solvent (0.005 μg/mL, corresponding to 0.01 mg/kg). Middle plots: soybeans spiked with 0.01 mg/kg of the pesticide. Lower plots: soybean blank extract.

Figure 1 shows the extracted ion chromatograms of representative pesticides in soybeans. No interfering peaks were detected in the extracted ion chromatograms of blank samples at the retention times of the target pesticides, which indicate the high selectivity of the method. The only exceptions were tridemorph in soybeans and peanuts. The interfering peaks were, however, less than 1/10 of the peak areas of the 0.005 μg/mL (corresponding to 0.01 mg/kg) standard solution of the target pesticides, conforming to the criteria of the Japanese validation guideline [21]. In addition, the retention times of the target pesticides in the matrices were found to be in good agreement with those in the solvent standard solutions (within ±0.02 min). Furthermore, the RSDs of retention times were <0.5% in brown rice, soybeans, and peanuts, except five pesticides (hexaconazole, isoprocarb, methidathion, pentoxazone, and quizalofop-ethyl) that showed low sensitivity.

The results of method validation revealed that LOQs, defined as the lowest concentration that can be quantified with satisfactory trueness values and precision, are 0.01 mg/kg for most pesticides (Table S1). MRLs of the target pesticides in brown rice, soybeans, and peanuts established in Japan are shown in Table S1. For pesticide/food combinations whose MRLs are not established, a uniform limit of 0.01 mg/kg is applied. As can be seen, MRLs are ≥0.01 mg/kg. Therefore, the proposed method exhibits sufficient sensitivity for regulatory purpose analysis.

### 3.3. Confirmation

**Figure 2:**
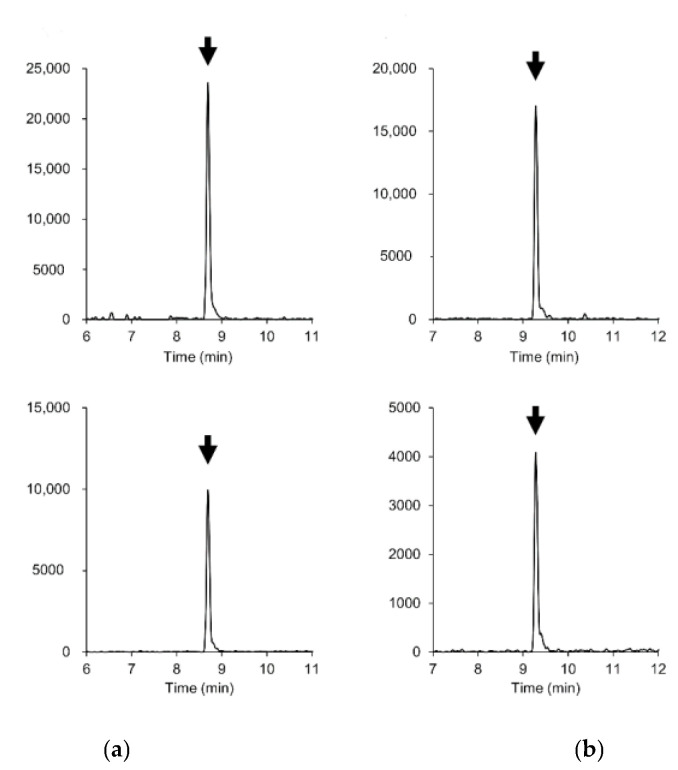
Extracted ion chromatograms (mass window ±5 mDa) of parent and fragment ions of (**a**) boscalid and (**b**) cyazofamid in 0.01 mg/kg-spiked soybean blank extract. Upper plots: [M + H]^+^ ((**a**) *m/z* 343.0399, (**b**) *m/z* 325.0521). Lower plots: fragment ions ((**a**) *m/z* 307.0633, (**b**) *m/z* 108.0114).

**Figure 3:**
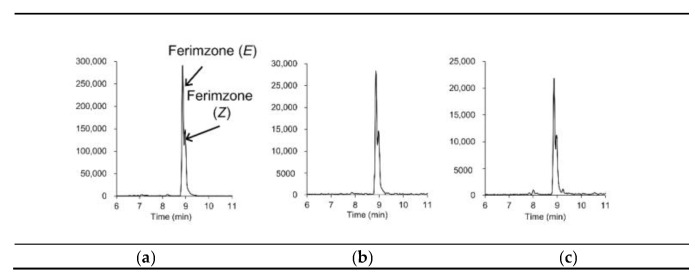
Extracted ion chromatograms (mass window ±5 mDa) of incurred ferimzone residue in a rice sample: (**a**) parent ion ([M+H]^+^, *m/z* 255.1604) and (**b**,**c**) its fragment ions ((**b**) *m/z* 132.0808, (**c**) *m/z* 124.0869).

For discriminating analytes from coeluting matrix components in complex foods at low concentrations, information on the exact mass and retention times of the target analytes may not be sufficient, even when using LC-Orbitrap-MS, which, compared to LC-TOF-MS, provides a high resolving power [19]. The EU guidelines [22] state that two ions, preferably a molecular adduct and at least one fragment ion, are required for accurate mass measurement by high-resolution MS. Hybrid HR-MS, such as QTOF-MS and QOrbitrap-MS, provide fragment-ion information via data dependent acquisition (DDA) and/or DIA [2,18]. In DDA, precursor ions are sequentially selected from full scans based on user-selected criteria (e.g., minimal intensity threshold, *m/z* values). In contrast, in DIA, all ionized compounds are subjected to fragmentation, and thus, DIA provides information regarding the fragment ions derived from all ions. In a previous study into pesticide residue analyses in vegetables and fruits using LC–QTOF-MS [14], we demonstrated DIA using the MS^E^ technique (Waters) [24], which provided full-scan data on both the molecular adduct (at low collision energy) and fragment (at high collision energy) ions in a single run, without selecting the precursor ion. In the study reported herein, we further applied the MS^E^ technique to brown rice, soybeans, and peanuts spiked at a level of 0.01 mg/kg. Figure 2 shows extracted ion chromatograms of molecular adduct and fragment ions from the soybean samples; Table 1 shows that the fragment ions could be detected at 0.005 μg/mL in the presence of the matrices. Among the 146 target pesticides, for 126 pesticides, we were able to detect one or more fragment ions; for 84 pesticides, we were able to detect one or more isotopic ions, and for 134 pesticides, we were able to detect one or more fragment ions and/or isotopic ions. Figure 3 shows extracted ion chromatograms of incurred ferimzone residue found in the rice sample used for validation in this study. As can be seen, the [M+H]^+^ (*m/z* 255.1604) for ferimzone together with its two fragments ions, i.e., [C_9_H_10_N]^+^ (*m/z* 132.0808) and [C_6_H_10_N_3_]^+^ (*m/z* 124.0869), were clearly detected, suggesting that the MS^E^ technique could be a useful tool for obtaining fragment ion information for confirmation purposes. However, because the sensitivities of the fragment ion peaks for several pesticides were low, more sensitive methods, such as LC-MS/MS in SRM mode, may be required for confirmation, especially for the pesticides that were shown to be detected with low sensitivities using the LC–QTOF-MS technique described in this study.

## 4. Conclusions

In this study, the multiresidue method using LC–QTOF-MS in full-scan acquisition mode was validated for the determination of 151 pesticides in cereal grains and legumes. Sufficiently high sensitivities were achieved at 0.005 μg/mL (corresponding to 0.01 mg/kg), with the exception of 5 of the 151 pesticides. Excellent results were obtained in terms of trueness, intra- and inter-day precision, and selectivity for most of the target pesticides at 0.01 mg/kg. The results revealed that the LC–QTOF-MS method offers reliable quantitative analysis of pesticide residues in cereal grains and legumes. In addition, we demonstrated the usefulness of the MS^E^ technique for obtaining information on fragment ions for confirmation. Although we were unable to detect several parent and fragment ions owing to low S/N at 0.01 mg/kg, the LC–QTOF-MS method was shown to be suitable for regulatory-purpose analysis for most of the target pesticides.

## Figures and Tables

**Table 1:** Elemental composition, retention time, and calculated exact mass of the target pesticides.

Compound	Retention Time (min)	Molecular Formula	Type of Ion	Calculated Exact Mass (*m/z*)	Fragment Ion 1		Fragment Ion 2	
					Elemental Composition	Calculated Exact Mass (*m/z)*	Elemental Composition	Calculated Exact Mass (*m/z*)
Acetamiprid	5.5	C_10_H_11_ClN_4_	[M+H]^+^	223.0745	C_6_H_5_ClN	126.0105	C_6_H_4_N	90.0338
Acetochlor	9.3	C_14_H_20_ClNO_2_	[M+H]^+^	270.1255	C_12_H_15_ClNO	224.0837	C_10_H_14_N	148.1121
Acibenzolar-*S*-methyl	9.1	C_8_H_6_N_2_OS_2_	[M+H]^+^	210.9994	C_6_H_4_N_2_S	136.0090		
Acrinathrin	11.0	C_26_H_21_F_6_NO_5_	[M+NH_4_]^+^	559.1662	C_13_H_9_O	181.0648		
Ametryn	8.8	C_9_H_17_N_5_S	[M+H]^+^	228.1277	C_6_H_12_N_5_S	186.0808	C_4_H_6_N_3_	96.0556
Anilofos	9.6	C_13_H_19_ClNO_3_PS_2_	[M+H]^+^	368.0305	C_4_H_8_O_3_PS_2_	198.9647	C_2_H_6_O_2_PS	124.9821
Aramite	10.4	C_15_H_23_ClO_4_S	[M+NH_4_]^+^	352.1344	C_13_H_19_O	191.1430		
Atrazine	8.1	C_8_H_14_ClN_5_	[M+H]^+^	216.1010	C_5_H_9_ClN_5_	174.0541	C_4_H_6_N_3_	96.0556
Azoxystrobin	8.7	C_22_H_17_N_3_O_5_	[M+H]^+^	404.1241	C_21_H_14_N_3_O_4_	372.0979	C_19_H_11_N_3_O_3_	329.0795
Benalaxyl	9.7	C_20_H_23_NO_3_	[M+H]^+^	326.1751	C_10_H_14_N	148.1121	C_12_H_18_NO_2_	208.1332
Bendiocarb	7.1	C_11_H_13_NO_4_	[M+H]^+^	224.0917	C_6_H_5_O_2_	109.0284		
Benzofenap	10.3	C_22_H_20_Cl_2_N_2_O_3_	[M+H]^+^	431.0924	C_8_H_9_	105.0699	C_8_H_7_O	119.0491
Bitertanol	9.8	C_20_H_23_N_3_O_2_	[M+H]^+^	338.1863				
Boscalid	8.7	C_18_H_12_Cl_2_N_2_O	[M+H]^+^	343.0399	C_18_H_12_ClN_2_O	307.0633	C_6_H_3_ClNO	139.9898
Bromacil	7.1	C_9_H_13_BrN_2_O_2_	[M+H]^+^	261.0233	C_5_H_6_BrN_2_O_2_	204.9607		
Buprofezin	10.4	C_16_H_23_N_3_OS	[M+H]^+^	306.1635	C_9_H_17_N_2_OS	201.1056	C_7_H_8_N	106.0651
Butafenacil	9.1	C_20_H_18_ClF_3_N_2_O_6_	[M+NH_4_]^+^	492.1144	C_13_H_7_ClF_3_N_2_O_3_	331.0092	C_8_H_3_ClNO_2_	179.9847
Cadusafos	10.0	C_10_H_23_O_2_PS_2_	[M+H]^+^	271.0950	C_2_H_8_O_2_PS_2_	158.9698	H_4_O_2_PS_2_	130.9385
Carbaryl	7.2	C_12_H_11_NO_2_	[M+H]^+^	202.0863				
Carpropamid	9.6	C_15_H_18_Cl_3_NO	[M+H]^+^	334.0527	C_8_H_8_Cl	139.0309	C_7_H_12_Cl_2_NO	196.0290
Chlorfenvinphos (*E, Z*)	9.7, 9.8	C_12_H_14_Cl_3_O_4_P	[M+H]^+^	358.9768	C_4_H_12_O_4_P	155.0468		
Chloridazon	5.6	C_10_H_8_ClN_3_O	[M+H]^+^	222.0429				
Chloroxuron	9.0	C_15_H_15_ClN_2_O_2_	[M+H]^+^	291.0895	C_3_H_6_NO	72.0444	C_9_H_12_N_2_O	164.0944
Chlorpyrifos	10.7	C_9_H_11_Cl_3_NO_3_PS	[M+H]^+^	349.9336	C_5_H_3_Cl_3_NO	197.9275	H_2_O_2_PS	96.9508
Chlorpyrifos methyl	10.1	C_7_H_7_Cl_3_NO_3_PS	[M+H]^+^	321.9023				
Chromafenozide	9.2	C_24_H_30_N_2_O_3_	[M+H]^+^	395.2329	C_11_H_11_O_2_	175.0754		
Clomeprop	10.4	C_16_H_15_Cl_2_NO_2_	[M+H]^+^	324.0553				
Cloquintocet mexyl	10.5	C_18_H_22_ClNO_3_	[M+H]^+^	336.1361	C_11_H_9_ClNO_3_	238.0265	C_10_H_7_ClNO	192.0211
Clothianidin	5.0	C_6_H_8_ClN_5_O_2_S	[M+H]^+^	250.0160	C_6_H_9_N_4_S	169.0542	C_4_H_3_ClNS	131.9669
Cumyluron	9.0	C_17_H_19_ClN_2_O	[M+H]^+^	303.1259	C_8_H_10_ClN_2_O	185.0476	C_7_H_6_Cl	125.0153
Cyanazine	6.9	C_9_H_13_ClN_6_	[M+H]^+^	241.0963	C_8_H_13_ClN_5_	214.0854	C_4_H_6_N_3_	96.0556
Cyazofamid	9.3	C_13_H_13_ClN_4_O_2_S	[M+H]^+^	325.0521	C_2_H_6_NO_2_S	108.0114		
Cycloprothrin	10.9	C_26_H_21_Cl_2_NO_4_	[M+NH_4_]^+^	499.1186				
Cyflufenamid	9.8	C_20_H_17_F_5_N_2_O_2_	[M+H]^+^	413.1283	C_12_H_12_F_5_N_2_O	295.0864	C_8_H_6_F_5_N_2_O	241.0395
Cyproconazole	8.8, 9.0	C_15_H_18_ClN_3_O	[M+H]^+^	292.1211	C_7_H_6_Cl	125.0153		
Cyprodinil	9.9	C_14_H_15_N_3_	[M+H]^+^	226.1339				
Daimuron	8.9	C_17_H_20_N_2_O	[M+H]^+^	269.1648	C_8_H_11_N_2_O	151.0866		
Deltamethrin	11.0	C_22_H_19_Br_2_NO_3_	[M+NH_4_]^+^	523.0049				
Diazinon	9.8	C_12_H_21_N_2_O_3_PS	[M+H]^+^	305.1083	C_5_H_15_NO_3_S	169.0767	H_2_O_2_PS	96.9508
Difenoconazole	9.7, 10.0	C_19_H_17_Cl_2_N_3_O_3_	[M+H]^+^	406.0720	C_13_H_9_Cl_2_O	251.0025	C_17_H_15_Cl_2_O_3_	337.0393
Diflubenzuron	9.3	C_14_H_9_ClF_2_N_2_O_2_	[M+H]^+^	311.0393	C_7_H_6_F_2_NO	158.0412	C_7_H_3_F_2_O	141.0146
Diflufenican	10.1	C_19_H_11_F_5_N_2_O_2_	[M+H]^+^	395.0813	C_13_H_7_F_3_NO_2_	266.0423	C_13_H_6_F_2_NO_2_	246.0361
Dimethirimol	7.8	C_11_H_19_N_3_O	[M+H]^+^	210.1601	C_8_H_14_NO	140.1070	C_5_H_8_NO	98.0600
Dimethoate	5.4	C_5_H_12_NO_3_PS_2_	[M+H]^+^	230.0069	C_4_H_8_O_3_PS_2_	198.9647	C_2_H_6_O_2_PS	124.9821
Dimethomorph (*E, Z*)	8.6, 8.8	C_21_H_22_ClNO_4_	[M+H]^+^	388.1310	C_17_H_14_ClO_3_	301.0626	C_9_H_9_O_3_	165.0546
Diuron	8.1	C_9_H_10_Cl_2_N_2_O	[M+H]^+^	233.0243	C_3_H_6_NO	72.0444	C_6_H_4_Cl_2_N	159.9715
Edifenphos	9.7	C_14_H_15_O_2_PS_2_	[M+H]^+^	311.0324	C_12_H_12_O_2_PS_2_	283.0011	C_6_H_5_S	109.0106
Epoxiconazole	9.2	C_17_H_13_ClFN_3_O	[M+H]^+^	330.0804	C_5_H_10_ClO	121.0415		
Ethion	10.6	C_9_H_22_O_4_P_2_S_4_	[M+H]^+^	384.9949	CH_4_O_2_PS_2_	142.9385	C_5_H_12_O_2_PS_2_	199.0011
Ethiprole	8.5	C_13_H_9_Cl_2_F_3_N_4_OS	[M+H]^+^	396.9899	C_11_H_4_Cl_2_F_3_N_4_S	350.9480	C_8_H_4_Cl_2_F_3_N_2_	254.9698
Etoxazole	10.8	C_21_H_23_F_2_NO_2_	[M+H]^+^	360.1770	C_7_H_3_F_2_O	141.0146	C_17_H_16_F_2_NO_2_	304.1144
Etrimfos	9.8	C_10_H_17_N_2_O_4_PS	[M+H]^+^	293.0719	C_8_H_14_N_2_O_4_PS	265.0406	C_2_H_6_O_2_PS	124.9821
Fenamidone	8.7	C_17_H_17_N_3_OS	[M+H]^+^	312.1165	C_15_H_14_N_3_	236.1182	C_6_H_6_N	92.0495
Fenamiphos	9.3	C_13_H_22_NO_3_PS	[M+H]^+^	304.1131				
Fenarimol	9.2	C_17_H_12_Cl_2_N_2_O	[M+H]^+^	331.0399	C_4_H_5_N_2_	81.0447		
Fenbuconazole	9.2	C_19_H_17_ClN_4_	[M+H]^+^	337.1215	C_7_H_6_Cl	125.0153	C_2_H_4_N_3_	70.0400
Fenobucarb	8.5	C_12_H_17_NO_2_	[M+H]^+^	208.1332				
Fenoxaprop ethyl	10.3	C_18_H_16_ClNO_5_	[M+H]^+^	362.0790	C_15_H_11_ClNO_3_	288.0422		
Fenoxycarb	9.5	C_17_H_19_NO_4_	[M+H]^+^	302.1387	C_3_H_6_NO_2_	88.0393	C_5_H_10_NO_2_	116.0706
Fenpropathrin	10.8	C_22_H_23_NO_3_	[M+H]^+^	350.1751				
Fenpropimorph	11.4	C_20_H_33_NO	[M+H]^+^	304.2635				
Ferimzone (*E, Z*)	8.9(*E*), 9.0(*Z*)	C_15_H_18_N_4_	[M+H]^+^	255.1604	C_9_H_10_N	132.0808	C_6_H_10_N_3_	124.0869
Fipronil	9.3	C_12_H_4_Cl_2_F_6_N_4_OS	[M+NH_4_]^+^	453.9725	C_11_H_5_Cl_2_F_3_N_4_OS	367.9508		
Flamprop methyl	9.0	C_17_H_15_ClFNO_3_	[M+H]^+^	336.0797	C_7_H_5_O	105.0335		
Fludioxonil	8.8	C_12_H_6_F_2_N_2_O_2_	[M+NH_4_]^+^	266.0736				
Flufenacet	9.1	C_14_H_13_F_4_N_3_O_2_S	[M+H]^+^	364.0737	C_8_H_7_FNO	152.0506	C_11_H_13_FNO	194.0976
Fluquinconazole	9.1	C_16_H_8_Cl_2_FN_5_O	[M+H]^+^	376.0163	C_14_H_6_Cl_2_FN_2_O	306.9836		
Fluridone	8.6	C_19_H_14_F_3_NO	[M+H]^+^	330.1100	C_19_H_14_F_2_NO	310.1038		
Fluvalinate	11.1	C_26_H_22_ClF_3_N_2_O_3_	[M+H]^+^	503.1344	C_13_H_9_O	181.0648		
Furametpyr	7.9	C_17_H_20_ClN_3_O_2_	[M+H]^+^	334.1317	C_6_H_6_ClN_2_O	157.0163	C_15_H_17_ClN_3_O	290.1055
Hexaconazole	9.7	C_14_H_17_Cl_2_N_3_O	[M+H]^+^	314.0821				
Hexaflumuron	10.1	C_16_H_8_Cl_2_F_6_N_2_O_3_	[M+H]^+^	460.9889	C_7_H_6_F_2_NO	158.0412		
Hexythiazox	10.6	C_17_H_21_ClN_2_O_2_S	[M+H]^+^	353.1085	C_9_H_11_ClN	168.0575	C_10_H_11_ClNOS	228.0244
Imazalil	9.6	C_14_H_14_Cl_2_N_2_O	[M+H]^+^	297.0556	C_11_H_9_Cl_2_N_2_O	255.0086		
Imibenconazole	10.4	C_17_H_13_Cl_3_N_4_S	[M+H]^+^	410.9999	C_7_H_6_Cl	125.0153	C_8_H_8_ClS	171.0030
Indanofan	9.3	C_20_H_17_ClO_3_	[M+H]^+^	341.0939	C_11_H_11_O_2_	175.0754		
Indoxacarb	10.0	C_22_H_17_ClF_3_N_3_O_7_	[M+H]^+^	528.0780	C_8_H_4_F_3_NO_2_	203.0189	C_9_H_7_F_3_NO_2_	218.0423
Iprovalicarb	9.1	C_18_H_28_N_2_O_3_	[M+H]^+^	321.2173				
Isoprocarb	7.9	C_11_H_15_NO_2_	[M+H]^+^	194.1176				
Isoxathion	10.0	C_13_H_16_NO_4_PS	[M+H]^+^	314.0610	C_7_H_5_O	105.0335	C_11_H_13_NO_4_PS	286.0297
Kresoxim methyl	9.6	C_18_H_19_NO_4_	[M+H]^+^	314.1387	C_15_H_12_NO	222.0913	C_16_H_11_O_2_	235.0754
Lactofen	10.3	C_19_H_15_ClF_3_NO_7_	[M+NH_4_]^+^	479.0827	C_14_H_6_ClF_3_NO_4_	343.9932	C_8_H_3_ClF_3_O_2_	222.9768
Linuron	8.7	C_9_H_10_Cl_2_N_2_O_2_	[M+H]^+^	249.0192	C_8_H_7_ClN_2_O	182.0241	C_6_H_4_Cl_2_N	159.9715
Lufenuron	10.5	C_17_H_8_Cl_2_F_8_N_2_O_3_	[M+H]^+^	510.9857	C_7_H_6_F_2_NO	158.0412		
Malathion	9.0	C_10_H_19_O_6_PS_2_	[M+H]^+^	331.0433	C_6_H_7_O_3_	127.0390	C_4_H_3_O_3_	99.0077
Mepanipyrim	9.4	C_14_H_13_N_3_	[M+H]^+^	224.1182	C_7_H_8_N	106.0651	C_13_H_11_N_3_	209.0947
Metalaxyl	8.0	C_15_H_21_NO_4_	[M+H]^+^	280.1543	C_13_H_18_NO_2_	220.1332	C_12_H_18_NO	192.1383
Methabenzthiazuron	8.1	C_10_H_11_N_3_OS	[M+H]^+^	222.0696	C_8_H_9_N_2_S	165.0481	C_7_H_6_N_2_S	150.0246
Methidathion	8.4	C_6_H_11_N_2_O_4_PS_3_	[M+H]^+^	302.9691				
Methiocarb	8.7	C_11_H_15_NO_2_S	[M+H]^+^	226.0896	C_8_H_9_O	121.0648	C_9_H_13_OS	169.0682
Metolachlor	9.4	C_15_H_22_ClNO_2_	[M+H]^+^	284.1412	C_14_H_19_ClNO	252.1150	C_12_H_18_N	176.1434
Monolinuron	7.7	C_9_H_11_ClN_2_O_2_	[M+H]^+^	215.0582	C_6_H_5_ClN	126.0105	C_8_H_8_N_2_O	148.0631
Myclobutanil	8.8	C_15_H_17_ClN_4_	[M+H]^+^	289.1215	C_7_H_6_Cl	125.0153		
Naproanilide	9.5	C_19_H_17_NO_2_	[M+H]^+^	292.1332	C_12_H_11_O	171.0804	C_8_H_10_N	120.0808
Napropamide	9.3	C_17_H_21_NO_2_	[M+H]^+^	272.1645	C_12_H_11_O	171.0804	C_13_H_11_O_2_	199.0754
Norflurazon	8.3	C_12_H_9_ClF_3_N_3_O	[M+H]^+^	304.0459	C_12_H_9_ClF_2_N_3_O	284.0397	C_7_H_5_F_3_N	160.0369
Novaluron	10.1	C_17_H_9_ClF_8_N_2_O_4_	[M+H]^+^	493.0196	C_7_H_6_F_2_NO	158.0412	C_7_H_3_F_2_O	141.0146
Oxadixyl	6.6	C_14_H_18_N_2_O_4_	[M+H]^+^	279.1339	C_12_H_15_N_2_O_2_	219.1128		
Oxaziclomefone	10.3	C_20_H_19_Cl_2_NO_2_	[M+H]^+^	376.0866	C_11_H_12_NO_2_	190.0863	C_10_H_9_O_2_	161.0597
Paclobutrazol	8.7	C_15_H_20_ClN_3_O	[M+H]^+^	294.1368	C_2_H_4_N_3_	70.0400		
Penconazole	9.5	C_13_H_15_Cl_2_N_3_	[M+H]^+^	284.0716	C_7_H_5_Cl_2_	158.9763		
Pencycuron	9.9	C_19_H_21_ClN_2_O	[M+H]^+^	329.1415				
Pentoxazone	10.3	C_17_H_17_ClFNO_4_	[M+H]^+^	354.0903				
Phenmedipham	8.3	C_16_H_16_N_2_O_4_	[M+H]^+^	301.1183	C_7_H_6_NO_2_	136.0393	C_8_H_10_NO_3_	168.0655
Phenthoate	9.6	C_12_H_17_O_4_PS_2_	[M+H]^+^	321.0379	C_9_H_12_O_2_PS_2_	247.0011		
Phosalone	9.8	C_12_H_15_ClNO_4_PS_2_	[M+H]^+^	367.9941	C_8_H_5_ClNO_2_	182.0003	C_7_H_5_ClN	138.0105
Phosphamidon	6.7	C_10_H_19_ClNO_5_P	[M+H]^+^	300.0762	C_8_H_13_ClNO	174.0680	C_2_H_8_O_4_P	127.0155
Piperonyl butoxide	10.6	C_19_H_30_O_5_	[M+NH_4_]^+^	356.2431	C_11_H_13_O_2_	177.0910		
Pirimicarb	7.9	C_11_H_18_N_4_O_2_	[M+H]^+^	239.1503	C_3_H_6_NO	72.0444		
Pirimiphos methyl	10.0	C_11_H_20_N_3_O_3_PS	[M+H]^+^	306.1036	C_9_H_14_N_3_	164.1182	C_5_H_6_N_3_	108.0556
Prochloraz	9.8	C_15_H_16_Cl_3_N_3_O_2_	[M+H]^+^	376.0381	C_12_H_13_Cl_3_NO_2_	308.0006	C_9_H_7_Cl_3_NO_2_	265.9537
Profenofos	10.3	C_11_H_15_BrClO_3_PS	[M+H]^+^	372.9424	C_6_H_6_BrClO_3_PS	302.8642	C_9_H_12_BrClO_3_PS	344.9111
Prometryn	9.3	C_10_H_19_N_5_S	[M+H]^+^	242.1434	C_4_H_8_N_5_S	158.0495	C_7_H_14_N_5_S	200.0964
Propachlor	8.1	C_11_H_14_ClNO	[M+H]^+^	212.0837	C_8_H_9_ClNO	170.0367		
Propanil	8.6	C_9_H_9_Cl_2_NO	[M+H]^+^	218.0134	C_6_H_6_ClN	127.0183	C_6_H_6_Cl_2_N	161.9872
Propaquizafop	10.4	C_22_H_22_ClN_3_O_5_	[M+H]^+^	444.1321	C_5_H_10_NO	100.0757		
Propargite	10.7	C_19_H_26_O_4_S	[M+NH_4_]^+^	368.1890				
Propiconazole	9.6	C_15_H_17_Cl_2_N_3_O_2_	[M+H]^+^	342.0771				
Propyzamide	8.9	C_12_H_11_Cl_2_NO	[M+H]^+^	256.0290	C_7_H_6_Cl_2_NO	189.9821	C_7_H_3_Cl_2_O	172.9555
Pyraclofos	9.8	C_14_H_18_ClN_2_O_3_PS	[M+H]^+^	361.0537	C_9_H_7_ClN_2_O_3_P	256.9877		
Pyraclostrobin	9.9	C_19_H_18_ClN_3_O_4_	[M+H]^+^	388.1059	C_10_H_12_NO_3_	194.0812		
Pyrazophos	10.1	C_14_H_20_N_3_O_5_PS	[M+H]^+^	374.0934	C_10_H_12_N_3_O_3_	222.0873	C_8_H_8_N_3_O_3_	194.0560
Pyriftalid	8.7	C_15_H_14_N_2_O_4_S	[M+H]^+^	319.0747	C_6_H_7_N_2_O_2_	139.0502	C_15_H_13_N_2_O_3_S	301.0641
Pyrimethanil	8.9	C_12_H_13_N_3_	[M+H]^+^	200.1182				
Pyriproxyfen	10.7	C_20_H_19_NO_3_	[M+H]^+^	322.1438	C_5_H_6_NO	96.0444	C_12_H_9_O_2_	185.0597
Quinalphos	9.7	C_12_H_15_N_2_O_3_PS	[M+H]^+^	299.0614	C_8_H_7_N_2_O	147.0553		
Quinoxyfen	10.7	C_15_H_8_Cl_2_FNO	[M+H]^+^	308.0040	C_15_H_8_ClFNO	272.0273	C_9_H_5_Cl_2_N	196.9794
Quizalofop ethyl	10.3	C_19_H_17_ClN_2_O_4_	[M+H]^+^	373.0950				
Simazine	7.3	C_7_H_12_ClN_5_	[M+H]^+^	202.0854	C_6_H_10_N_3_	124.0869	C_4_H_7_ClN_3_	132.0323
Simeconazole	9.0	C_14_H_20_FN_3_OSi	[M+H]^+^	294.1432	C_2_H_4_N_3_	70.0400		
Spinosyn A	11.4	C_41_H_65_NO_10_	[M+H]^+^	732.4681	C_8_H_16_NO	142.1226		
Spinosyn D	11.7	C_42_H_67_NO_10_	[M+H]^+^	746.4838	C_8_H_16_NO	142.1226		
Spiroxamine	10.4, 10.5	C_18_H_35_NO_2_	[M+H]^+^	298.2741	C_8_H_18_NO	144.1383	C_6_H_14_N	100.1121
Tebuconazole	9.5	C_16_H_22_ClN_3_O	[M+H]^+^	308.1524	C_2_H_4_N_3_	70.0400		
Tebufenpyrad	10.4	C_18_H_24_ClN_3_O	[M+H]^+^	334.1681	C_4_H_6_ClN_2_	117.0214		
Tebuthiuron	7.2	C_9_H_16_N_4_OS	[M+H]^+^	229.1118	C_7_H_14_N_3_S	172.0903	C_3_H_6_N_3_S	116.0277
Teflubenzuron	10.4	C_14_H_6_Cl_2_F_4_N_2_O_2_	[M+H]^+^	380.9815				
Terbutryn	9.4	C_10_H_19_N_5_S	[M+H]^+^	242.1434	C_6_H_12_N_5_S	186.0808	C_5_H_8_N_5_	138.0774
Tetrachlorvinphos	9.4	C_10_H_9_Cl_4_O_4_P	[M+H]^+^	366.9036	C_2_H_8_O_4_P	127.0155	C_8_H_3_Cl_3_	203.9295
Tetraconazole	9.1	C_13_H_11_Cl_2_F_4_N_3_O	[M+H]^+^	372.0288	C_7_H_5_Cl_2_	158.9763	C_2_H_4_N_3_	70.0400
Thiacloprid	6.0	C_10_H_9_ClN_4_S	[M+H]^+^	253.0309	C_6_H_5_ClN	126.0105	C_6_H_4_N	90.0338
Tolfenpyrad	10.5	C_21_H_22_ClN_3_O_2_	[M+H]^+^	384.1473	C_14_H_13_O	197.0961	C_6_H_10_ClN_2_	145.0527
Triadimefon	8.9	C_14_H_16_ClN_3_O_2_	[M+H]^+^	294.1004	C_11_H_14_ClO	197.0728		
Triadimenol	8.9	C_14_H_18_ClN_3_O_2_	[M+H]^+^	296.1160	C_2_H_4_N_3_	70.0400		
Triazophos	9.2	C_12_H_16_N_3_O_3_PS	[M+H]^+^	314.0723	C_8_H_8_N_3_O	162.0662	C_7_H_7_N_2_	119.0604
Tricyclazole	6.4	C_9_H_7_N_3_S	[M+H]^+^	190.0433	C_8_H_7_N_2_S	163.0324	C_7_H_6_NS	136.0215
Tridemorph	11.9, 12.3	C_19_H_39_NO	[M+H]^+^	298.3104				
Trifloxystrobin	10.1	C_20_H_19_F_3_N_2_O_4_	[M+H]^+^	409.1370	C_9_H_7_F_3_N	186.0525	C_11_H_12_NO_3_	206.0812
Triflumizole	10.1	C_15_H_15_ClF_3_N_3_O	[M+H]^+^	346.0929	C_12_H_12_ClF_3_NO	278.0554		
Triflumuron	9.8	C_15_H_10_ClF_3_N_2_O_3_	[M+H]^+^	359.0405	C_7_H_7_ClNO	156.0211	C_7_H_4_ClO	138.9945
Triticonazole	9.1	C_17_H_20_ClN_3_O	[M+H]^+^	318.1368	C_2_H_4_N_3_	70.0400		

**Table 2:** Trueness and intra- and inter-day precision of the target pesticides.

Compound	Brown Rice			Soybeans			Peanuts		
	Trueness (%)	Intra-Day Precision (relative standard deviation (RSD)%)	Inter-Day Precision (RSD%)	Trueness (%)	Intra-Day Precision (RSD%)	Inter-Day Precision (RSD%)	Trueness (%)	Intra-Day Precision (RSD%)	Inter-Day Precision (RSD%)
Acetamiprid	87	13	13	77	11	17	85	7	11
Acetochlor	77	1	4	84	5	8	89	4	5
Acibenzolar *S*-methyl	82	5	10	80	8	8	82	4	4
Acrinathrin	72	9	9	78	5	20	62	4	4
Ametryn	86	4	4	87	6	6	89	2	2
Anilofos	84	3	3	84	5	7	90	2	2
Aramite	80	3	6	83	6	7	71	6	6
Atrazine	87	3	3	89	5	7	91	2	2
Azoxystrobin	88	3	3	90	4	6	92	2	2
Benalaxyl	86	4	4	88	7	8	91	2	2
Bendiocarb	81	6	9	86	6	14	87	4	6
Benzofenap	84	3	5	83	6	9	86	3	3
Bitertanol	84	3	18	80	4	23	82	3	6
Boscalid	86	3	3	86	4	6	91	2	3
Bromacil	81	2	2	83	3	7	87	3	3
Buprofezin	82	4	5	75	10	10	73	2	2
Butafenacil	86	3	3	88	4	7	93	1	2
Cadusafos	81	2	3	78	6	9	84	4	4
Carbaryl	86	3	5	89	4	6	92	2	3
Carpropamid	82	3	3	82	7	8	88	2	2
Chlorfenvinphos (*E, Z*)	85	2	3	85	6	9	90	2	3
Chloridazon	77	5	5	80	4	5	88	3	3
Chloroxuron	80	4	6	84	5	7	92	2	2
Chlorpyrifos	83	3	5	77	8	9	75	5	5
Chlorpyrifos methyl	77	7	12	83	5	10	80	8	9
Chromafenozide	73	5	6	83	5	9	84	4	8
Clomeprop	79	4	6	74	6	9	70	6	6
Cloquintocet mexyl	88	2	3	86	7	11	85	2	3
Clothianidin	71	7	7	77	3	5	81	2	2
Cumyluron	77	3	3	87	3	6	88	4	4
Cyanazine	85	3	4	87	5	7	85	4	4
Cyazofamid	71	4	5	78	3	6	80	4	5
Cycloprothrin	61	8	14	47	9	17	70	3	5
Cyflufenamid	81	2	3	81	5	10	88	2	3
Cyproconazole	76	3	3	82	5	8	88	1	3
Cyprodinil	84	2	2	42	37	37	78	1	2
Daimuron	71	6	12	91	4	5	91	6	7
Deltamethrin	85	2	8	81	7	13	38	3	11
Diazinon	86	5	5	86	5	6	87	3	3
Difenoconazole	75	3	3	76	4	10	80	2	3
Diflubenzuron	74	5	7	73	5	8	86	2	3
Diflufenican	78	3	4	77	5	8	77	3	3
Dimethirimol	72	5	8	79	5	6	79	2	3
Dimethoate	78	3	5	82	3	6	88	3	3
Dimethomorph (*E, Z*)	83	3	3	90	5	7	91	1	3
Diuron	84	3	4	86	4	7	91	2	3
Edifenphos	83	3	3	81	7	7	85	3	3
Epoxiconazole	65	6	11	71	6	8	84	3	4
Ethion	80	2	3	79	5	8	81	2	3
Ethiprole	85	3	4	86	4	5	87	2	3
Etoxazole	71	5	11	65	11	11	70	4	4
Etrimfos	81	4	5	84	2	7	84	3	4
Fenamidone	84	4	4	86	4	8	89	2	2
Fenamiphos	74	14	16	64	25	25	90	2	3
Fenarimol	74	2	3	73	3	7	71	3	3
Fenbuconazole	73	3	3	74	2	5	84	2	2
Fenobucarb	86	6	8	83	7	8	89	5	5
Fenoxaprop ethyl	77	3	3	77	5	7	80	5	5
Fenoxycarb	81	1	5	85	4	9	91	1	3
Fenpropathrin	61	7	8	36	14	19	60	3	7
Fenpropimorph	88	4	6	80	4	6	25	19	27
Ferimzone	—^1^	—^1^	—^1^	89	4	5	91	2	3
Fipronil	66	5	9	70	7	10	79	2	2
Flamprop methyl	77	2	3	88	4	9	91	3	3
Fludioxonil	79	5	5	85	5	12	88	2	4
Flufenacet	80	3	4	84	3	11	86	3	4
Fluquinconazole	84	4	8	79	3	8	88	3	4
Fluridone	89	4	4	89	4	7	93	2	2
Fluvalinate	44	8	30	49	12	19	22	10	15
Furametpyr	86	3	3	88	4	7	92	2	2
Hexaconazole	—^2^	—^2^	—^2^	—^2^	—^2^	—^2^	—^2^	—^2^	—^2^
Hexaflumuron	77	10	13	83	7	9	80	10	10
Hexythiazox	76	3	4	55	7	14	70	3	4
Imazalil	78	4	4	81	7	9	91	4	4
Imibenconazole	66	3	9	65	8	13	60	10	10
Indanofan	75	4	4	71	4	6	77	6	11
Indoxacarb	84	2	5	85	4	8	85	2	3
Iprovalicarb	84	4	5	87	4	7	92	2	2
Isoprocarb	—^2^	—^2^	—^2^	—^2^	—^2^	—^2^	—^2^	—^2^	—^2^
Isoxathion	86	2	2	80	7	8	89	10	11
Kresoxim methyl	83	3	9	85	8	13	88	6	6
Lactofen	82	2	6	76	6	9	82	2	3
Linuron	85	3	3	86	4	6	90	2	3
Lufenuron	73	6	11	81	5	9	84	6	8
Malathion	78	4	8	87	6	12	92	4	4
Mepanipyrim	84	2	4	80	6	6	85	3	3
Metalaxyl	90	3	3	90	4	7	92	3	3
Methabenzthiazuron	84	4	4	87	6	6	89	3	3
Methidathion	—^2^	—^2^	—^2^	—^2^	—^2^	—^2^	—^2^	—^2^	—^2^
Methiocarb	83	8	8	88	7	10	89	4	4
Metolachlor	85	2	3	81	5	8	89	2	3
Monolinuron	86	5	7	86	6	6	91	3	3
Myclobutanil	85	3	4	87	5	7	89	1	2
Naproanilide	83	3	3	81	5	7	88	2	2
Napropamide	86	4	5	86	4	7	91	2	3
Norflurazon	86	3	4	86	2	6	92	2	2
Novaluron	79	8	8	79	6	10	80	3	3
Oxadixyl	88	7	8	90	4	4	92	2	3
Oxaziclomefone	86	2	6	82	8	10	83	6	6
Paclobutrazol	83	3	3	83	5	8	84	4	4
Penconazole	80	3	3	81	6	11	85	1	2
Pencycuron	84	3	3	83	5	9	86	2	2
Pentoxazone	—^2^	—^2^	—^2^	—^2^	—^2^	—^2^	—^2^	—^2^	—^2^
Phenmedipham	70	3	8	71	4	4	80	11	11
Phenthoate	82	3	9	79	7	8	88	4	4
Phosalone	81	3	4	81	5	9	85	2	4
Phosphamidon	84	4	4	89	4	4	90	2	2
Piperonyl butoxide	84	2	3	80	8	8	77	7	10
Pirimicarb	86	5	5	89	6	6	88	2	2
Pirimiphos methyl	89	2	3	85	6	7	84	3	3
Prochloraz	81	3	3	83	5	9	86	2	2
Profenofos	83	4	5	79	7	10	82	2	3
Prometryn	85	2	3	83	5	6	88	2	2
Propachlor	78	3	4	81	6	6	87	3	3
Propanil	81	5	5	85	5	10	90	3	4
Propaquizafop	83	3	4	82	5	9	83	3	3
Propargite	77	4	9	73	5	7	78	4	4
Propiconazole	81	3	3	82	6	8	86	1	2
Propyzamide	81	4	5	86	4	8	89	4	4
Pyraclofos	85	2	3	85	5	7	88	2	2
Pyraclostrobin	85	4	4	83	6	7	91	1	1
Pyrazophos	87	2	4	85	8	8	84	5	6
Pyriftalid	87	4	4	88	4	6	93	2	2
Pyrimethanil	95	3	4	85	5	5	85	3	3
Pyriproxyfen	79	1	2	71	8	10	71	2	3
Quinalphos	86	3	6	82	8	8	88	1	2
Quinoxyfen	72	2	2	61	9	13	60	3	4
Quizalofop ethyl	—^2^	—^2^	—^2^	—^2^	—^2^	—^2^	—^2^	—^2^	—^2^
Simazine	85	3	4	86	5	5	92	2	2
Simeconazole	79	2	4	82	5	5	84	7	9
Spinosyn A	62	5	12	70	4	8	58	4	4
Spinosyn D	75	2	5	71	4	6	79	3	3
Spiroxamine	83	3	15	74	2	10	73	2	6
Tebuconazole	78	3	3	81	5	8	86	1	2
Tebufenpyrad	80	2	4	73	7	11	72	3	3
Tebuthiuron	79	2	3	86	3	5	88	2	2
Teflubenzuron	74	6	13	70	15	15	72	8	13
Terbutryn	86	3	3	84	5	6	88	2	2
Tetrachlorvinphos	83	1	2	85	4	7	91	2	3
Tetraconazole	79	2	2	81	3	6	89	1	2
Thiacloprid	81	3	4	85	4	4	89	2	2
Tolfenpyrad	79	1	5	80	6	9	79	3	3
Triadimefon	87	5	5	86	4	5	91	2	2
Triadimenol	78	8	8	89	8	12	89	3	4
Triazophos	79	7	14	85	5	11	89	5	7
Tricyclazole	—^1^	—^1^	—^1^	79	4	5	80	1	2
Tridemorph	71	10	16	65	6	23	36	6	16
Trifloxystrobin	87	3	3	85	4	7	87	2	3
Triflumizole	80	5	8	76	5	10	80	5	5
Triflumuron	72	3	3	79	7	8	84	3	3
Triticonazole	79	4	5	83	5	6	88	1	2

^1^ Not evaluated due to residue being found in the sample. ^2^ Not evaluated due to low sensitivity.

**Table 3:** Matrix effects of the target pesticides in brown rice, soybeans, and peanuts.

Compound	Brown Rice	Soybeans	Peanuts
Acetamiprid	0.99	0.88	1.03
Acetochlor	0.89	0.94	1.01
Acibenzolar *S*-methyl	0.98	1.01	0.98
Acrinathrin	0.70	0.72	0.68
Ametryn	0.98	0.99	1.02
Anilofos	0.94	0.96	1.01
Aramite	0.85	0.91	0.93
Atrazine	1.00	0.97	1.01
Azoxystrobin	0.98	0.98	1.00
Benalaxyl	0.95	0.98	0.99
Bendiocarb	0.92	1.01	1.02
Benzofenap	0.91	0.97	0.98
Bitertanol	0.89	0.93	0.93
Boscalid	0.95	0.97	0.98
Bromacil	0.95	0.94	0.97
Buprofezin	0.96	0.94	0.96
Butafenacil	0.94	0.97	0.99
Cadusafos	0.95	0.92	0.97
Carbaryl	0.99	0.97	1.04
Carpropamid	0.94	0.94	0.98
Chlorfenvinphos (*E, Z*)	0.92	0.96	0.99
Chloridazon	0.99	0.95	1.02
Chloroxuron	0.91	0.97	1.01
Chlorpyrifos	0.96	0.93	1.02
Chlorpyrifos methyl	1.05	0.95	0.92
Chromafenozide	0.85	0.90	0.90
Clomeprop	0.89	0.91	0.93
Cloquintocet mexyl	0.97	0.96	1.00
Clothianidin	0.98	0.88	1.00
Cumyluron	0.89	0.95	0.99
Cyanazine	0.98	0.96	1.00
Cyazofamid	0.80	0.95	0.97
Cycloprothrin	0.71	0.81	0.89
Cyflufenamid	0.90	0.91	0.97
Cyproconazole	0.87	0.93	0.98
Cyprodinil	0.99	0.98	0.98
Daimuron	0.81	1.05	1.04
Deltamethrin	0.63	0.70	0.53
Diazinon	0.99	0.97	0.99
Difenoconazole	0.86	0.86	0.89
Diflubenzuron	0.86	0.89	0.96
Diflufenican	0.84	0.92	0.90
Dimethirimol	0.99	0.99	1.01
Dimethoate	0.97	0.94	1.01
Dimethomorph (*E, Z*)	0.96	0.97	0.98
Diuron	0.95	0.96	1.00
Edifenphos	0.94	0.96	1.00
Epoxiconazole	0.69	0.91	0.98
Ethion	0.89	0.93	0.94
Ethiprole	0.96	0.95	0.96
Etoxazole	0.91	0.90	0.97
Etrimfos	1.01	0.94	1.01
Fenamidone	0.95	0.95	0.99
Fenamiphos	0.81	0.82	1.00
Fenarimol	0.85	0.87	0.87
Fenbuconazole	0.80	0.91	0.94
Fenobucarb	0.99	0.93	0.98
Fenoxaprop ethyl	0.89	0.89	0.95
Fenoxycarb	0.91	0.93	0.99
Fenpropathrin	0.71	0.73	0.93
Fenpropimorph	1.08	0.98	0.81
Ferimzone	—^1^	1.02	1.00
Fipronil	0.67	0.87	0.95
Flamprop methyl	0.85	0.97	0.99
Fludioxonil	0.84	0.91	0.95
Flufenacet	0.91	0.93	0.93
Fluquinconazole	0.95	0.87	0.92
Fluridone	0.99	0.99	1.01
Fluvalinate	0.53	0.52	0.38
Furametpyr	0.99	0.96	1.00
Hexaconazole	—^2^	—^2^	—^2^
Hexaflumuron	0.84	0.91	0.84
Hexythiazox	0.81	0.77	0.94
Imazalil	0.98	0.98	1.04
Imibenconazole	0.79	0.85	0.86
Indanofan	0.80	0.94	0.97
Indoxacarb	0.91	0.93	0.94
Iprovalicarb	0.92	0.95	0.98
Isoprocarb	—^2^	—^2^	—^2^
Isoxathion	0.92	0.93	0.94
Kresoxim methyl	0.90	0.91	0.95
Lactofen	0.86	0.91	0.96
Linuron	0.94	0.95	0.99
Lufenuron	0.81	0.87	0.97
Malathion	0.88	0.96	1.00
Mepanipyrim	0.96	0.97	0.99
Metalaxyl	1.01	0.97	1.00
Methabenzthiazuron	0.98	0.98	0.99
Methidathion	—^2^	—^2^	—^2^
Methiocarb	0.94	0.96	0.97
Metolachlor	0.92	0.95	0.98
Monolinuron	1.02	1.01	0.99
Myclobutanil	0.94	0.95	0.97
Naproanilide	0.90	0.93	0.97
Napropamide	0.96	0.99	0.99
Norflurazon	0.98	0.96	1.00
Novaluron	0.82	0.92	0.88
Oxadixyl	1.00	1.01	1.00
Oxaziclomefone	0.89	0.91	0.96
Paclobutrazol	0.93	0.94	0.98
Penconazole	0.91	0.90	0.95
Pencycuron	0.96	0.96	0.95
Pentoxazone	—^2^	—^2^	—^2^
Phenmedipham	0.97	0.95	1.09
Phenthoate	0.93	0.96	1.00
Phosalone	0.88	0.93	0.96
Phosphamidon	1.00	1.00	1.01
Piperonyl butoxide	0.90	0.95	0.95
Pirimicarb	1.02	1.00	1.00
Pirimiphos methyl	0.99	0.99	0.99
Prochloraz	0.86	0.92	0.97
Profenofos	0.92	0.94	0.96
Prometryn	0.99	0.98	1.00
Propachlor	1.02	0.96	0.99
Propanil	0.92	0.95	0.99
Propaquizafop	0.91	0.96	0.99
Propargite	0.81	0.87	0.94
Propiconazole	0.90	0.94	0.96
Propyzamide	0.94	0.97	0.99
Pyraclofos	0.95	0.97	0.97
Pyraclostrobin	0.97	0.98	0.98
Pyrazophos	0.96	0.96	0.93
Pyriftalid	0.99	0.98	1.00
Pyrimethanil	1.02	0.99	1.00
Pyriproxyfen	0.88	0.91	0.94
Quinalphos	0.93	0.93	0.99
Quinoxyfen	0.83	0.80	0.89
Quizalofop ethyl	—^2^	—^2^	—^2^
Simazine	0.98	0.97	1.04
Simeconazole	0.87	0.93	0.98
Spinosyn A	0.72	0.83	0.63
Spinosyn D	0.90	0.90	0.92
Spiroxamine	0.97	0.96	0.97
Tebuconazole	0.88	0.93	0.96
Tebufenpyrad	0.88	0.94	0.95
Tebuthiuron	0.99	1.00	1.00
Teflubenzuron	0.77	0.91	0.90
Terbutryn	0.99	0.97	1.01
Tetrachlorvinphos	0.92	0.96	0.99
Tetraconazole	0.86	0.91	0.96
Thiacloprid	0.98	0.94	1.00
Tolfenpyrad	0.87	0.94	0.98
Triadimefon	0.92	0.98	0.98
Triadimenol	0.87	0.92	0.93
Triazophos	0.90	0.96	0.93
Tricyclazole	—^1^	0.99	1.02
Tridemorph	1.05	1.03	0.98
Trifloxystrobin	0.96	0.96	0.96
Triflumizole	0.85	0.90	0.90
Triflumuron	0.80	0.90	0.98
Triticonazole	0.88	0.92	0.97

^1^ Not evaluated due to residue being found in the sample. ^2^ Not evaluated due to low sensitivity.

## Data Availability

The data presented in this study are available in the article or supplementary material.

## Supplementary Materials

The following are available online at https://www.mdpi.com/2304-8158/10/1/78/s1, Table S1: MRLs set in Japan and LOQs of the target pesticides. Table S2: Results of method validation at MRL.
