# Supplementary material for: Quantitative and Confirmatory Analysis of Pesticide Residues in Cereal Grains and Legumes by Liquid Chromatography–Quadrupole-Time-of-Flight Mass Spectrometry
Source: Foods. 2021 Jan 3;10(1):78. doi: 10.3390/foods10010078 (PMC7823789; doi:10.3390/foods10010078)
Supplement: Supplementary file 1 [file foods-10-00078-s001.pdf]

Supplementary Material

Quantitative and Confirmatory Analysis of Pesticide Residues in Cereal Grains and Legumes by Liquid Chromatography–Quadrupole-Time-of-Flight Mass Spectrometry

Shizuka Saito-Shida, Satoru Nemoto, Hiroshi Akiyama

Division of Foods, National Institute of Health Sciences

*Corresponding author. E-mail: shizsaito@nihs.go.jp

Table S1. MRLs set in Japan and LOQs of the target pesticides.

	Brown rice		Soybeans		Peanuts	
Compound	MRL	LOQ	MRL	LOQ	MRL	LOQ
Acetamiprid	—[a)]	0.01	0.3	0.01	0.2	0.01
Acetochlor	—[a)]	0.01	1	0.01	—[a)]	0.01
Acibenzolar *S*-methyl	0.1	0.01	—[a)]	0.01	—[a)]	0.01
Acrinathrin	—[a)]	0.01	—[a)]	0.01	—[a)]	—[d)]
Ametryn	—[a)]	0.01	—[a)]	0.01	—[a)]	0.01
Anilofos	—[a)]	0.01	—[a)]	0.01	—[a)]	0.01
Aramite	—[a)]	0.01	—[a)]	0.01	—[a)]	0.01
Atrazine	—[a)]	0.01	0.1	0.01	0.1	0.01
Azoxystrobin	0.2	0.01	0.5	0.01	0.2	0.01
Benalaxyl	0.05	0.01	0.05	0.01	0.05	0.01
Bendiocarb	0.02	0.01	—[a)]	0.01	—[a)]	0.01
Benzofenap	0.05	0.01	—[a)]	0.01	—[a)]	0.01
Bitertanol	—[a)]	0.01	0.2	0.01	0.1	0.01
Boscalid	—[a)]	0.01	3	0.01	1	0.01
Bromacil	—[a)]	0.01	—[a)]	0.01	—[a)]	0.01
Buprofezin	0.5	0.01	0.02	0.01	—[a)]	0.01
Butafenacil	—[a)]	0.01	—[a)]	0.01	—[a)]	0.01
Cadusafos	—[a)]	0.01	0.01	0.01	—[a)]	0.01
Carbaryl	1	0.01	0.2	0.01	0.05	0.01
Carpropamid	1	0.01	—[a)]	0.01	—[a)]	0.01
Chlorfenvinphos (*E*, *Z*)	0.05	0.01	0.02	0.01	0.05	0.01
Chloridazon	—[a)]	0.01	—[a)]	0.01	—[a)]	0.01
Chloroxuron	—[a)]	0.01	—[a)]	0.01	—[a)]	0.01
Chlorpyrifos	0.1	0.01	0.3	0.01	0.2	0.01
Chlorpyrifos methyl	0.1	0.01	0.05	0.01	0.05	0.01
Chromafenozide	0.2	0.01	0.5	0.01	—[a)]	0.01
Clomeprop	0.02	0.01	—[a)]	0.01	—[a)]	0.01
Cloquintocet mexyl	—[a)]	0.01	—[a)]	0.01	—[a)]	0.01
Clothianidin	1	0.01	0.1	0.01	0.02	0.01
Cumyluron	0.1	0.01	—[a)]	0.01	—[a)]	0.01

Cyanazine	—[a)]	0.01	—[a)]	0.01	—[a)]	0.01
Cyazofamid	0.05	0.01	0.3	0.01	—[a)]	0.01
Cycloprothrin	0.05	—[d)]	—[a)]	—[d)]	—[a)]	0.01
Cyflufenamid	—[a)]	0.01	—[a)]	0.01	—[a)]	0.01
Cyproconazole	0.1	0.01	0.05	0.01	0.02	0.01
Cyprodinil	—[a)]	0.01	0.1	0.1[e)]	—[a)]	0.01
Daimuron	0.1	0.01	—[a)]	0.01	—[a)]	0.01
Deltamethrin	—[a)]	0.01	1	0.01	—[a)]	—[d)]
Diazinon	—[a)]	0.01	0.05	0.01	0.02	0.01
Difenoconazole	0.2	0.01	0.2	0.01	0.01	0.01
Diflubenzuron	—[a)]	0.01	0.05	0.01	0.1	0.01
Diflufenican	—[a)]	0.01	0.05	0.01	—[a)]	0.01
Dimethirimol	—[a)]	0.01	—[a)]	0.01	—[a)]	0.01
Dimethoate	1	0.01	1	0.01	1	0.01
Dimethomorph (*E*, *Z*)	—[a)]	0.01	0.2	0.01	—[a)]	0.01
Diuron	0.05	0.01	0.05	0.01	0.05	0.01
Edifenphos	0.2	0.01	—[a)]	0.01	—[a)]	0.01
Epoxiconazole	—[a)]	—[d)]	0.05	0.01	0.05	0.01
Ethion	—[a)]	0.01	—[a)]	0.01	—[a)]	0.01
Ethiprole	0.2	0.01	0.2	0.01	—[a)]	0.01
Etoxazole	—[a)]	0.01	—[a)]	—[d)]	—[a)]	0.01
Etrimfos	—[a)]	0.01	—[a)]	0.01	—[a)]	0.01
Fenamidone	—[a)]	0.01	—[a)]	0.01	—[a)]	0.01
Fenamiphos	0.02	0.01	0.05	0.05[e)]	0.05	0.01
Fenarimol	—[a)]	0.01	0.02	0.01	0.02	0.01
Fenbuconazole	—[a)]	0.01	0.2	0.01	0.1	0.01
Fenobucarb	1	0.01	—[a)]	0.01	—[a)]	0.01
Fenoxaprop ethyl	0.05	0.01	0.1	0.01	0.05	0.01
Fenoxycarb	0.05	0.01	0.05	0.01	0.05	0.01
Fenpropathrin	—[a)]	—[d)]	0.1	0.1[e)]	0.01	—[d)]
Fenpropimorph	0.3	0.01	0.05	0.01	0.05	—[d)]
Ferimzone	2	—[b)]	—[a)]	0.01	—[a)]	0.01
Fipronil	0.01	—[d)]	—[a)]	0.01	—[a)]	0.01
Flamprop methyl	—[a)]	0.01	—[a)]	0.01	—[a)]	0.01

Fludioxonil	0.02	0.01	0.5	0.01	—[a)]	0.01
Flufenacet	—[a)]	0.01	0.1	0.01	—[a)]	0.01
Fluquinconazole	—[a)]	0.01	—[a)]	0.01	—[a)]	0.01
Fluridone	—[a)]	0.01	—[a)]	0.01	—[a)]	0.01
Fluvalinate	—[a)]	—[d)]	0.05	—[d)]	—[a)]	—[d)]
Furametpyr	0.5	0.01	—[a)]	0.01	—[a)]	0.01
Hexaconazole	—[a)]	—[c)]	—[a)]	—[c)]	—[a)]	—[c)]
Hexaflumuron	—[a)]	0.01	—[a)]	0.01	—[a)]	0.01
Hexythiazox	—[a)]	0.01	0.5	0.5[e)]	—[a)]	0.01
Imazalil	0.05	0.01	0.02	0.01	0.02	0.01
Imibenconazole	—[a)]	—[d)]	0.3	0.3[e)]	0.1	—[d)]
Indanofan	0.05	0.01	—[a)]	0.01	—[a)]	0.01
Indoxacarb	—[a)]	0.01	5	0.01	0.02	0.01
Iprovalicarb	—[a)]	0.01	—[a)]	0.01	—[a)]	0.01
Isoprocarb	—[a)]	—[c)]	—[a)]	—[c)]	—[a)]	—[c)]
Isoxathion	—[a)]	0.01	0.02	0.01	0.02	0.01
Kresoxim methyl	—[a)]	0.01	—[a)]	0.01	—[a)]	0.01
Lactofen	—[a)]	0.01	0.01	0.01	0.01	0.01
Linuron	0.1	0.01	0.5	0.01	0.5	0.01
Lufenuron	—[a)]	0.01	0.05	0.01	—[a)]	0.01
Malathion	0.1	0.01	2	0.01	8	0.01
Mepanipyrim	—[a)]	0.01	—[a)]	0.01	—[a)]	0.01
Metalaxyl	0.1	0.01	0.05	0.01	0.1	0.01
Methabenzthiazuron	0.05	0.01	0.05	0.01	—[a)]	0.01
Methidathion	0.02	0.02[e)]	0.1	0.1[e)]	0.1	—[d)]
Methiocarb	0.05	0.01	0.05	0.01	0.05	0.01
Metolachlor	0.1	0.01	0.2	0.01	0.2	0.01
Monolinuron	—[a)]	0.01	—[a)]	0.01	—[a)]	0.01
Myclobutanil	—[a)]	0.01	0.3	0.01	—[a)]	0.01
Naproanilide	—[a)]	0.01	—[a)]	0.01	—[a)]	0.01
Napropamide	—[a)]	0.01	—[a)]	0.01	—[a)]	0.01
Norflurazon	—[a)]	0.01	—[a)]	0.01	0.05	0.01
Novaluron	—[a)]	0.01	0.1	0.01	—[a)]	0.01
Oxadixyl	0.1	0.01	5	0.01	5	0.01

Oxaziclomefone	0.05	0.01	—[a)]	0.01	—[a)]	0.01
Paclobutrazol	0.02	0.01	—[a)]	0.01	—[a)]	0.01
Penconazole	0.05	0.01	0.05	0.01	0.05	0.01
Pencycuron	0.3	0.01	—[a)]	0.01	—[a)]	0.01
Pentoxazone	0.05	0.05[e)]	—[a)]	—[c)]	—[a)]	—[c)]
Phenmedipham	—[a)]	0.01	—[a)]	0.01	—[a)]	0.01
Phenthoate	0.05	0.01	0.05	0.01	—[a)]	0.01
Phosalone	—[a)]	0.01	—[a)]	0.01	—[a)]	0.01
Phosphamidon	—[a)]	0.01	—[a)]	0.01	—[a)]	0.01
Piperonyl butoxide	24	0.01	0.2	0.01	1	0.01
Pirimicarb	0.3	0.01	1	0.01	—[a)]	0.01
Pirimiphos methyl	0.2	0.01	0.1	0.01	1	0.01
Prochloraz	2	0.01	0.1	0.01	0.1	0.01
Profenofos	—[a)]	0.01	—[a)]	0.01	—[a)]	0.01
Prometryn	0.1	0.01	0.1	0.01	0.02	0.01
Propachlor	—[a)]	0.01	—[a)]	0.01	—[a)]	0.01
Propanil	0.2	0.01	—[a)]	0.01	—[a)]	0.01
Propaquizafop	—[a)]	0.01	0.05	0.01	0.05	0.01
Propargite	—[a)]	0.01	0.3	0.01	0.1	0.01
Propiconazole	0.1	0.01	2	0.01	0.2	0.01
Propyzamide	—[a)]	0.01	—[a)]	0.01	—[a)]	0.01
Pyraclofos	—[a)]	0.01	—[a)]	0.01	—[a)]	0.01
Pyraclostrobin	—[a)]	0.01	0.05	0.01	0.05	0.01
Pyrazophos	—[a)]	0.01	—[a)]	0.01	—[a)]	0.01
Pyriftalid	0.02	0.01	—[a)]	0.01	—[a)]	0.01
Pyrimethanil	—[a)]	0.01	—[a)]	0.01	—[a)]	0.01
Pyriproxyfen	—[a)]	0.01	0.2	0.01	—[a)]	0.01
Quinalphos	—[a)]	0.01	0.05	0.01	0.05	0.01
Quinoxyfen	—[a)]	0.01	—[a)]	—[d)]	—[a)]	—[d)]
Quizalofop ethyl	—[a)]	—[c)]	—[a)]	—[c)]	—[a)]	—[c)]
Simazine	—[a)]	0.01	—[a)]	0.01	—[a)]	0.01
Simeconazole	0.1	0.01	0.2	0.01	—[a)]	0.01
Spinosyn A	0.1	—[d)]	0.02	0.01	0.02	—[d)]
Spinosyn D	0.1	0.01	0.02	0.01	0.02	0.01

Spiroxamine	—a)	0.01	—a)	0.01	—a)	0.01
Tebuconazole	0.05	0.01	0.2	0.01	0.2	0.01
Tebufenpyrad	—a)	0.01	—a)	0.01	—a)	0.01
Tebuthiuron	—a)	0.01	—a)	0.01	—a)	0.01
Teflubenzuron	—a)	0.01	0.05	0.01	—a)	0.01
Terbutryn	—a)	0.01	—a)	0.01	—a)	0.01
Tetrachlorvinphos	—a)	0.01	—a)	0.01	—a)	0.01
Tetraconazole	—a)	0.01	0.2	0.01	—a)	0.01
Thiacloprid	0.02	0.01	—a)	0.01	—a)	0.01
Tolfenpyrad	—a)	0.01	—a)	0.01	—a)	0.01
Triadimefon	0.3	0.01	0.2	0.01	0.2	0.01
Triadimenol	0.5	0.01	0.2	0.01	0.2	0.01
Triazophos	—a)	0.01	0.05	0.01	0.05	0.01
Tricyclazole	3	—b)	—a)	0.01	—a)	0.01
Tridemorph	0.05	0.01	0.1	—d)	0.1	—d)
Trifloxystrobin	2	0.01	0.08	0.01	0.05	0.01
Triflumizole	0.05	0.01	—a)	0.01	—a)	0.01
Triflumuron	0.05	0.01	0.02	0.01	0.02	0.01
Triticonazole	0.05	0.01	—a)	0.01	—a)	0.01

a) Uniform limit of 0.01 mg/kg is applied
b) Not evaluated because the residue is found in the sample.
c) Low sensitivity for monitoring MRL
d) Did not satisfy the criteria of trueness and/or precision required by the Japanese [21] and EU [22] method validation guidelines at MRL or lower concentration
e) Results of method validation are shown in Table S2

Table S2. Results of method validation at MRL.

Pesticide	Food	MRL	Spiked level	Trueness (%)	Intra-day precision (RSD%)	Inter-day precision (RSD%)
Methidathion	Brown rice	0.02	0.02	89	5	5
Pentoxazone	Brown rice	0.05	0.05	76	8	8
Cyprodinil	Soybeans	0.1	0.1	81	5	7
Fenamiphos	Soybeans	0.05	0.05	88	2	2
Fenpropathrin	Soybeans	0.1	0.1	70	12	14
Hexythiazox	Soybeans	0.5	0.5	73	8	8
Imibenconazole	Soybeans	0.3	0.3	81	7	7
Methidathion	Soybeans	0.1	0.1	90	4	4
